# Bilateral vocal fold immobility: diagnosis and treatment

**DOI:** 10.1590/S1808-86942011000500010

**Published:** 2015-10-22

**Authors:** José Antonio Pinto, Luciana Ballester de Mello Godoy, Valéria Wanderley Pinto Brandão Marquis, Thiago Branco Sonego, Carolina de Farias Aires Leal

**Affiliations:** 1 Otorhinolaryngologist. Director/Head; 2 Medical doctor, otorhinolaryngologist, assistant physician at the São Paulo Otorhinolaryngology and Head & Neck Surgery Unit; 3 Medical doctor, otorhinolaryngologist, assistant physician at the São Paulo Otorhinolaryngology and Head & Neck Surgery Unit; 4 Medical resident of the São Paulo Otorhinolaryngology and Head & Neck Surgery Unit; 5 Medical resident of the São Paulo Otorhinolaryngology and Head & Neck Surgery Unit

**Keywords:** glottis, paralysis, Vocal Cord Paralysis, vocal cords

## Abstract

**Abstract:**

Vocal fold immobility may be due to bilateral neurogenic paralysis, cricoarytenoid joint fixation, laryngeal synechiae, or posterior glottic stenosis. Treatment aims to establish a patent airway and preserve the function of the glottic sphincter and voice quality.

**Objetives:**

To analyze the diagnostic and therapeutic approaches in cases of bilateral vocal fold immobility seen at our unit.

**Materials and Methods:**

A retrospective study of 35 patient registries at our unit with a diagnosis of bilateral vocal fold immobility; the etiology and treatment results were evaluated.

**Results:**

Among the patients, 18 (51.4%) were cases of bilateral vocal fold palsy, and 17 (48,6%) were cases of posterior glottic stenosis. Patients with bilateral palsy underwent unilateral subtotal arytenoidectomy, and patients with stenosis were treated with the microtrapdoor flap technique, subtotal arytenoidectomy, and/or posterior cricoidotomy (Rethi).

**Conclusion:**

Bilateral vocal fold immobility is a potentially fatal condition; it is essential to differentiate vocal fold palsy from fixation to choose the appropriate treatment. Subtotal arytenoidectomy with microscopy is our surgery of choice for treating bilateral paralysis; the technique for treating stenosis depends on the amount of stenosis.

## INTRODUCTION

Vocal fold immobility is the term that describes restricted movement of vocal folds secondary to mechanical fixation or neurological involvement. Mobility of the vocal folds may be decreased or absent, and it may be unilateral or bilateral. From the standpoint of the etiology, choice of treatment, and prognosis, it is important to differentiate between hypomobility and immobility, as well as unilateral or bilateral involvement^1^.

There are two forms by which patients may present bilateral vocal fold immobility: existing stridor for weeks or months that worsens rapidly to dyspnea or progressive and gradual dyspnea in the course of a few months^2^, usually with no significant changes in voice quality.

The diagnosis and treatment of bilateral vocal fold immobility has been studied often in laryngology during the past few decades; several studies have described approaches that rehabilitate the larynx with a high success rate^3^.

Bilateral vocal fold immobility is a potentially fatal condition; it needs to be diagnosed promptly and accurately, and treated appropriately^2^. Bilateral vocal fold immobility may be caused by bilateral neurogenic palsy, fixation of the cricoarythenoid joint, laryngeal synechiae, or posterior glottic stenosis^2^. The differential diagnosis is based on the medical history, fibronasopharyngolaryngoscope findings, and laryngeal electromyography. In a few cases, the diagnosis is only possible by inspecting and palpating the larynx by microlaryngoscopy. Specific causes may be surgical trauma, post-intubation trauma, cancer, neurologic conditions, inflammatory diseases, and psychogenic causes^4^.

The treatment of bilateral vocal fold immobility aims to reestablish a patent airway, to preserve glottic sphincter function, and to maintain voice quality^5^. Existing surgical options are tracheotomy, total arythenoidectomy, subtotal arythenoidectomy, transverse cordectomy, vocal fold lateralization^6^, and open and reinnervation techniques^7^.

## OBJECTIVE

The purpose of this study was to analyze the diagnostic methods and therapeutic approaches in cases of bilateral vocal fold immobility seen at our unit.

## MATERIALS AND METHODS

The institutional review board assessed and approved this study (protocol no. 114/010. This was a retrospective quantitative study consisting of a review of patient registries diagnosed with bilateral vocal fold immobility by fibronasolaryngoscopy at our unit from 1992 to 2007. The cases were classified according to the etiology: (1) due to neurologic involvement (palsy); and (2) due to a decreased diameter of the posterior glottis (posterior glottic stenosis), in which case it was classified according to Bogdasarian & Olson^8^ (Table 1).

**Table 1:** Classification of Posterior Glottic Stenosis (Bogdasarian & Olson^8^).

TYPE I	Glottis – interarytenoid scar, normal posterior commissure
TYPE II	Interarytenoid scar and posterior commissure scar
TYPE III	Posterior commissure scar involving a cricoarytenoid joint
TYPE IV	Posterior commissure scar involving both cricoarytenoid joints

The etiology was defined based on the clinical history, the physical examination, and diagnostic tests such as electroneuromyography (EMG) and computed tomography (CT), if needed; it was compared with data in the available literature.

The type of treatment was analyzed according to the etiology and the degree and type of upper airway narrowing. Success was defined as a patent airway with preservation of the glottic sphincter function and voice quality.

## RESULTS

The sample comprised 35 patients with a diagnosis of bilateral vocal fold immobility at our unit from 1992 to 2007. Of this total, 18 cases (51.4%) presented bilateral vocal fold palsy, and 17 cases (48.6%) had posterior glottic stenosis.

Of the 18 patients with bilateral vocal fold palsy, four were male and 14 were female. The mean age was 45 years, ranging from 18 to 65 years. The etiology of bilateral vocal fold immobility was as follows: after thyroidectomy in 16 patients (88.9%), extrinsic compression of the mediastinum by a rhinopharyngeal lymphoepithelioma with lung metastasis in one patient (5.55%), and recurrence following resection of a mediastinal paraganglioma one patient (5.55%).

Among the patients with bilateral vocal fold palsy, laryngoscopy showed bilateral vocal fold immobility in adduction in 17 cases (94.4%), of which only four had tracheotomies. These patients underwent laryngeal microsurgery under general anesthesia to perform CO_2_ laser subtotal unilateral arytenoidectomy (Remacle)^9^ preserving a small posterior shell-shaped portion of the arytenoid cartilage. Granulation tissues formed in the arytenoid resection bed in two patients, which required surgical reexploration. Transitory aspiration occurred in four patients, and resolved spontaneously. Previously tracheostomized patients were decannulated on average 40 days after surgery. Only the patient with mediastinal paraganglioma had vocal fold immobility in abduction, and underwent type I thyroplasty.

**Figure 1:**
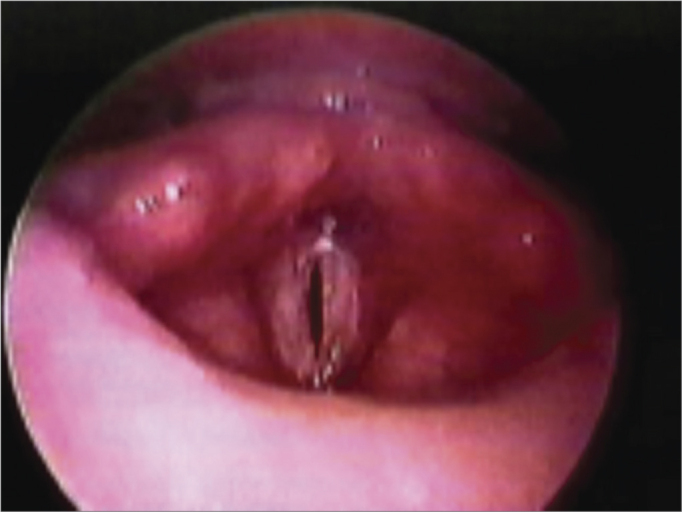
Posterior glottic stenosis grade III.

**Graph 1:**
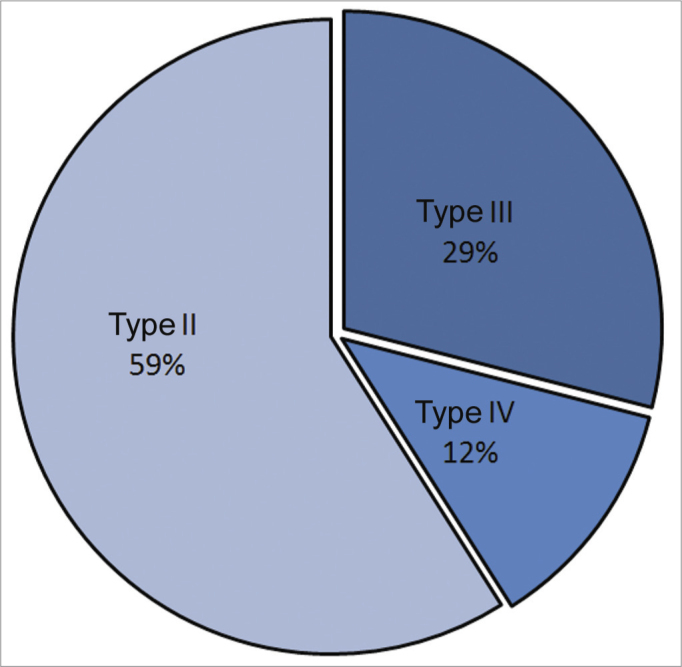
Type of posterior glottic stenosis in the series (Bogdasarian and Olson classification^8^).

Of 17 patients with posterior glottic stenosis (Table 2), 13 were male and four were female. The mean age was 44.7 years, ranging from 9 to 74 years. The etiology of stenosis was as follows: bilateral vocal fold immobility after prolonged orotracheal intubation in 15 patients (88.2%), after radiotherapy for laryngeal squamous cell carcinoma in one patient (5.9%), and after removal of a large laryngeal papilloma in one patient (5.9%). Posterior glottic stenosis was classified according to Bogdasarian & Olson^8^ (Table 1). It was type II in 10 cases (58.8%), type III in five cases (29.4%) (Figure 1), and type IV in two cases (11.8%) (Graph 1).

**Table 2:** Posterior Glottic Stenosis – Patient Data.

Patient	Age	S	Etiology	Type	Procedure	Follow-up (days/decannulation)
1-FMF	18	M	Intubation	II	MTDF	40 – decannulated
2- RA	24	F	Intubation	II	MTDF	74 – decannulated
3- JMA	12	M	Intubation	II	MTDF	66 – decannulated
4- LA	19	M	Intubation	II	MTDFU	61 – decannulated
5- MFM	36	F	Intubation	II	MTDFU	54 – decannulated
6- RM	28	M	Intubation	II	MTDF	53 – decannulated
7- ACL	11	M	Papillomatosis	II	MTDFU	47 – decannulated
8- HA	69	M	Intubation	III	MTDFU + AST	40 – decannulated
9- JHD	60	M	Intubation	II	MTDFU	39 – decannulated
10-ADM	13	M	Intubation	IV	MTDF+RETHI+MOLDE	35 – decannulated
11- JCI	15	M	Intubation	II	MTDFU	28 – decannulated
12- LRS	9	M	Intubation	III	MTDF+AST	21 – decannulated
13-MCS	19	F	Intubation	III	MTDF+AST	16 – decannulated
14- JMP	23	M	Intubation	III	MTDF+AST	11 – decannulated
15- JPF	28	F	Intubation	II	MTDFU	11 – decannulated
16-PML	74	M	Intubation	IV	AST+AT+RETHI+MOLDE	40 – decannulated
17- AG	61	M	RXT	III	MTDFU+AST	40 – decannulated

S = Sex

F = Female

M = Male

MTDF = Microtrapdoor Flap

MTDFU = Unilateral Microtrapdoor Flap

AST = Subtotal Arytenoidectomy

RETHI = Rethi's Technique (posterior cricoidotomy)

**Figure 2:**
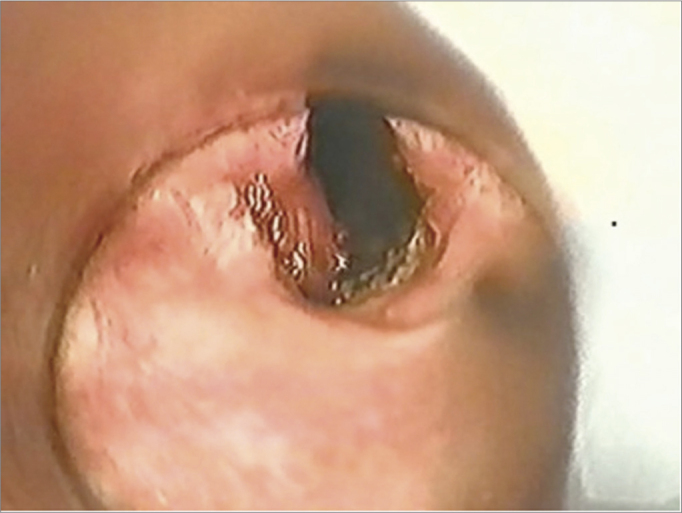
Unilateral microtrapdoor flap: posterior glottic stenosis grade III.

The microtrapdoor flap technique first described by Dedo & Sooy^10^ was used initially in eight patients (47%) (Figure 2) with posterior glottic stenosis. In this technique, an incision is made on the superior surface of the stenosis, the submucosa is dissected with CO_2_ laser to create a bipediculated lateral mucous flap, and the underlying scar tissue is removed. A variant of the microtrapdoor flap was used in eight patients (47%); in these cases, a vertical incision was made on the vocal process on one side and extended through a horizontal incision on the posterior interarytenoid surface to the other side, thereby creating a crescent moon-shaped unilaterally based mucous flap. The submucosal scar is easily vaporized by CO_2_ laser (0.25 mm microspot, superpulse mode, 5 watts). The mucous flap is thinned and placed over the open surface on one side and fixed with fibrin gel on the site (Tissucol^®^). This is the unilateral microtrapdoor flap technique.

Subtotal arytenoidectomy associated with the microtrapdoor flap was done in five patients (29.4%) with posterior glottic stenosis involving one of the cricothyroid joints, as described by Remacle.^9^

Decannulation was only possible after laryngofissure, posterior cricoidotomy, removal of scar tissue, placement of a cartilage graft and molding (Rethi's technique)^11^ in one patient (6%) with severe posterior glottic stenosis (Type IV) that had undergone microtrapdoor flap surgery. Subtotal arytenoidectomy, followed by total arytenoidectomy, as described by Ossoff^12^,^13^, was done in a second procedure; the arytenoid cartilage was removed, the muscle and vocal process were removed with laryngofissure and posterior cricoidotomy (Rethi's technique)^11^, and laryngeal molding was done (and removed 18 days later) in one patient (6%) with type IV posterior glottic stenosis.

The perception of voice in these patients according to the RASAT score was as follows:

- patients undergoing partial arytenoidectomy at first had moderate soprosity that regressed with speech therapy;
- the quality of voice did not worsen in patients undergoing the microtrapdoor flap procedure;
- patients undergoing more extensive procedures (Rethi + molding) had persisting moderate roughness and soprosity.

All patients had acceptable voice communication with mild to moderate degrees of dysphonia.

## DISCUSSION

Bilateral vocal fold immobility in adduction reduces the volume of the glottic space, and consequently increases airway resistance, which induces persistent dyspnea that worsens with exercise and upper airway inflammatory conditions. An accurate diagnosis and appropriate treatment is needed as this condition may progress to acute respiratory failure.

Bilateral vocal fold immobility may be diagnosed by fibronasopharyngolaryngoscopy, which will show lack of movement of both vocal folds. It may result from palsy of the recurrent laryngeal nerve or from posterior glottic stenosis. These conditions may present similar findings and the clinical history with endoscopy may not always differentiate these diseases. At this moment, laryngeal EMG is indicated for the etiological and differential diagnosis^14^. The action potential of thyroarytenoid muscles was evaluated by EMG in this study. Paralysis was excluded if the action potential of both thyroarytenoid muscles was unaltered; in these cases, the diagnosis was posterior glottic stenosis. Recurrent laryngeal nerve palsy was diagnosed if the action potential was abnormal.

Most studies on the etiology of bilateral vocal fold immobility have suggested that post-thyroidectomy bilateral vocal fold palsy is the most common cause^15^, ^16^, ^17^, ^18^. (Table 3). Feehery^19^ has shown that few papers have been published on this topic since 1980. His comparative study of the etiologic factors reported before and after 1980 showed a significant increase in cases of bilateral vocal fold immobility due to trauma (non-surgical and postsurgical intubation not due to thyroidectomy) and extrinsic compression by neoplasms (27% post-trauma, 21% due to neoplasms, 11% post-thyroidectomy)^19^. Rosenthal et al.^20^ studied the etiology of bilateral vocal fold immobility in the past 20 years also showed a lower incidence of bilateral palsy by iatrogenic causes in surgery. In our study, 16 of 35 patients (45.7%) had post-thyroidectomy bilateral vocal fold immobility, and 15 patients (42.8%) had this condition after prolonged intubation, which is comparable to recent papers on this topic.

**Table 3:** Etiology of bilateral vocal fold immobility (percentage %).

Etiology	Parnell^15^ 1970	Bulteau^16^ 1973	Maisel^17^ 1974	Tucker^18^ 1979	Feehery^19^ 2003	Pinto^25^ 2007
Thyroidectomy	78.6	37.5	40.7	45.6	10.7	45.7
Extralaryngeal tumor / Extrinsic compression	7.1	12.5	7.4	7.8	21.3	2.85
Mediastinal surgery	0	0	0	0	0	2.85
Intubation / Trauma	0	0	31.5	30	26.7	42.8
Neurologic	1.9	0	7.4	5.6	18.7	0
Idiopathic	0.6	50	3.7	0	13.3	0
Radiotherapy	0	0	0	0	0	2.9
Others	0	0	9.3	11.1	9.3	2.9
Total	14	8	54	180	75	35

Several surgical procedures have been proposed for the treatment of bilateral vocal fold immobility due to bilateral vocal fold palsy; these include external and endoscopic procedures^21^, ^22^, ^23^, ^24^, ^25^. Endoscopic approaches started with arytenoidectomy by electrocautery (Thornell)^26^ and CO_2_ laser total arytenoidectomy (Ossoff)^12^. Another conservative technique^25^, ^26^, ^27^, ^28^, ^29^ is subtotal arytenoidectomy (Remacle)^9^.

All of the patients in our sample with bilateral vocal fold immobility due to bilateral vocal fold palsy in adduction underwent subtotal arytenoidectomy (Remacle's technique)^9^. The success rate was 100% - all patients had patent airways. There were no post-operative complications except for transitory aspiration that resolved spontaneously, and a granuloma in one patient that was excised surgically.

Treatment of posterior glottic stenosis is still difficult; there are several available procedures and techniques, including endoscopic dilatation^30^, corticosteroids injected into lesions^8^, laryngofissure with posterior cricoidotomy^11^, excision of scars with placement of skin or mucosal grafts, and placement of different types of molds^10^,^31^,^32^.

We initially used the microtrapdoor flap technique bilaterally, according to Dedo & Sooy's^10^ original technique, in eight patients with a larger amount of interarytenoid mucous tissue. Subtotal arytenoidectomy was also done in five of these patients because of unilateral cricoarytenoid joint anquilosis. In two patients with severe posterior glottic stenosis (type IV) – one of which had been operated with the microtrapdoor flap technique and the other with subtotal arytenoidectomy – deccanulation was only possible after laryngofissure and posterior cricoidotomy with resection of scar tissue, graft placement, and molding (Rethi's technique^11^).

**Figure 3:**
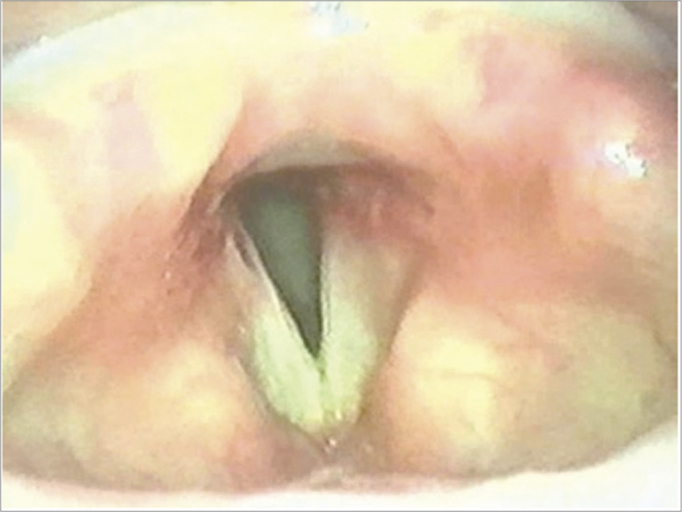
Post-operation – unilateral microtrapdoor flap.

The unilateral microtrapdoor flap technique was used in another eight cases of posterior glottic stenosis, as the incision facilitates dissection and better exposure of the stenotic scar. This technique was effective in 100% of cases; it was associated with subtotal arytenoidectomy in two of the type III posterior glottic stenosis cases (Figure 3).

An ideal treatment of bilateral vocal fold immobility has not been found in spite of advances in surgery and laryngology materials. Current techniques restore airway patency at the cost of possible worse sphincter function and voice quality. Research on laryngeal reinnervation has yielded improved muscle tone, less atrophy and vocal fold bowing, but no mobility gains^33^. Another promising line of research is of implantable stimulators, which maintain mobility and voice patterns, but as yet have yielded results only in experimental studies^34^.

Because this was a retrospective study, it is difficult to make accurate short/long term quantitative and qualitative analyses. However, the results are similar to those in the literature; further studies are needed to carefully evaluate these data.

## CONCLUSION

Bilateral vocal fold immobility is a potentially fatal condition; for this reason, accurate and prompt diagnosis followed by adequate treatment is mandatory. It is essential to differentiate palsy from vocal fold fixation in cases of bilateral vocal fold immobility so that the treatment is selected accordingly. EMG is indicated for this purpose.

Subtotal arytenoidectomy with a microscope is our choice of surgery for the treatment of bilateral vocal fold palsy in adduction. The choice of surgery in posterior glottic stenosis cases depends on the degree of stenosis (microtrapdoor flap and unilateral microtrapdoor flap in isolation or associated with subtotal arytenoidectomy and/or laryngofissure with posterior cricoidotomy – Rethi's technique).
